# How Right-Leaning Media Coverage of COVID-19 Facilitated the Spread of Misinformation in the Early Stages of the Pandemic in the U.S.

**DOI:** 10.1017/S0008423920000396

**Published:** 2020-05-01

**Authors:** Matt Motta, Dominik Stecula, Christina Farhart

**Affiliations:** 1 Department of Political Science, Oklahoma State University, 210 Murray Hall, Stillwater, OK 74078, USA; 2 Annenberg Public Policy Center, University of Pennsylvania, 202 S 36 St. Philadelphia, PA 19104, USA; 3 Centre for Public Opinion and Political Representation, Simon Fraser University, 8888 University Drive, Burnaby, BC V5A 1S6, Canada; 4 Department of Political Science, Carleton College, Willis Hall #415, Northfield, MN 55057, USA

## Abstract

We have yet to know the ultimate global impact of the novel coronavirus pandemic. However, we do know that delays, denials and misinformation about COVID-19 have exacerbated its spread and slowed pandemic response, particularly in the U.S. (e.g., Abutaleb et al., 2020).

## Introduction

We have yet to know the ultimate global impact of the novel coronavirus pandemic. However, we do know that delays, denials and misinformation about COVID-19 have exacerbated its spread and slowed pandemic response, particularly in the U.S. (e.g., Abutaleb et al., 2020).

While the role that misinformation played in slowing the federal government's response to COVID-19 is well-understood, less is known about why *Americans* might accept misinformation about the virus and how misinformation might affect trust in public health experts.

Polling from the early stages of the pandemic suggests that many Americans are misinformed about COVID-19. In early March 2020, a poll conducted by YouGov and The Economist found that 13 per cent of Americans believed the coronavirus was a hoax, 49 per cent believed the coronavirus was manmade, and 44 per cent believed the threat of the coronavirus was being exaggerated for political reasons (Economist, 2020). However, while COVID-19 misinformation is prevalent, it is not necessarily bipartisan. A March 1 Civiqs poll found 68 per cent of Democrats were moderately or extremely concerned about COVID-19, but only 21 per cent of Republicans expressed moderate or extreme concern (Badger and Quealy, 2020). Another Quinnipiac University poll released early in March found that roughly 6 in 10 Republican voters were not especially concerned that the coronavirus would disrupt their lives (Quinnipiac University/Poll, 2020; Russonello, 2020a). Further, there have been considerable partisan gaps with respect to how people were behaviorally responding to the crisis, for example, washing their hands, working from home, or changing their travel plans (Stecula, 2020).

We expect that variation in media coverage of the pandemic in its early stages may help explain these partisan differences. Some American media, particularly popular right-leaning outlets and pundits, spouted hoaxes and conspiracy theories behind the pandemic: Sean Hannity said the virus was a fraud by the “deep state” trying to spread panic, manipulate the economy, and suppress dissent; Rush Limbaugh suggested the virus was a plot hatched by the Chinese to harm the U.S. economy; and Fox Business anchor Trish Regan told viewers that the worry over coronavirus “is yet another attempt to impeach the president” (Peters and Grynbaum, 2020). As denial and disinformation exploded on right-leaning media outlets, many conservative elites correspondingly downplayed concern about the virus (Abutaleb et al., 2020; Badger and Quealy, 2020; Peters and Grynbaum, 2020; Russonello, 2020b; Warzel, 2020).

Consistent with this view, polling data from mid-March revealed that only 38 per cent of Fox News viewers were worried about coronavirus, compared to 72 per cent of national newspaper readers or 71 per cent of CNN viewers.

Previous academic research has demonstrated that people accept factually incorrect information as true if it originates from trusted sources or affirms their political and social worldviews (Kahan, 2017). Considerable evidence also suggests that political identity leads people to engage in motivated conspiracy endorsement impugning their political rivals (Flynn et al., 2017; Miller et al., 2016). These motivations may be amplified in an environment where the pandemic is highly politicized and trusted opinion leaders also endorse dubious COVID claims (Stecula, 2020). As a result, we believe that even seemingly innocuous denials or false claims from relied-upon media sources may lead individuals either into a false sense of security or lead others to ignore government recommendations.

The spread of misinformation about COVID-19 could be particularly problematic if misinformed people are subsequently less likely to trust advice from medical professionals. Previous research has found that misinformation about vaccine safety is associated with increased skepticism about the role medical professionals play in the policy-making process (Motta et al. 2018) and also with noncompliance with expert-backed health behaviors (such as wearing sunscreen or vaccinating children) (see Oliver and Wood, 2014).

Thus, we suspect that the highly partisan nature of early media coverage of the coronavirus pandemic had important public health consequences. The relative prominence of COVID misinformation shared by right-leaning media may have contributed to the spread of misinformation about COVID and subsequently undermined support for information from public health experts.

In this article, we show that right-leaning broadcast and cable media (for example, Fox News, Breitbart) regularly discussed misinformation about COVID-19 during the early stages of the pandemic. Further, nationally representative survey data suggest that people who *consumed* right-leaning media during that time were more likely to endorse COVID-19 misinformation. We find that misinformed people were more likely to believe that the CDC exaggerated COVID's health risks, suggesting that media coverage of the virus in the early stages of the pandemic may have had important public health consequences.

## Material and Methods

**Figure 1.**
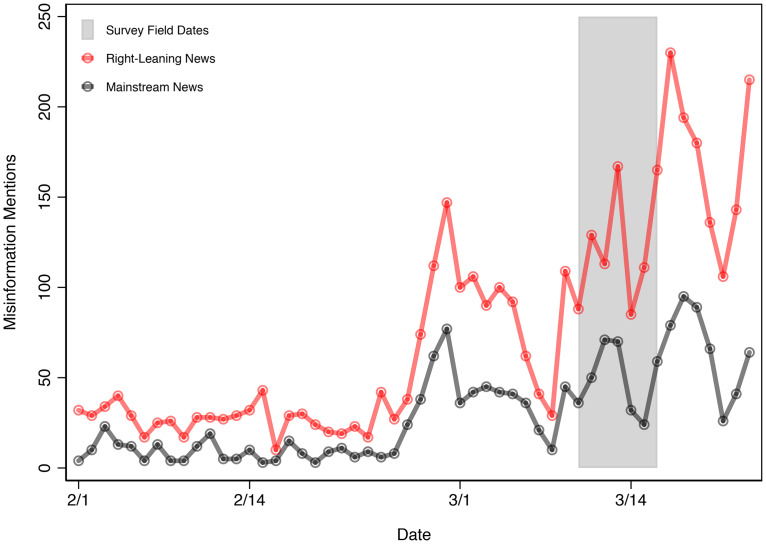
Prevalence of COVID-19 misinformation on right-leaning vs. mainstream media (February 1–March 23, 2020).

Note: Story counts are derived from queries of the MediaCloud Explorer database. Search terms included (“coronavirus” OR “covid”) and (“made in a lab” OR “Big Pharma” OR “George Soros” OR “hoax” OR “conspiracy” OR “bioweapon” OR “not real” OR “existing vaccine”). Please refer to the Methods section in the online Appendix for additional information about MediaCloud and our search protocol.

We gathered mentions of COVID misinformation from MediaCloud. We searched for key terms and phrases associated with coronavirus misinformation, such as the claim that COVID-19 was designed in a lab or that a vaccine already exists. These two claims mirror how we measure misinformation in our public opinion data (which we discuss shortly). The exact search protocol is outlined in the online Appendix and is summarized in Figure 1.

We examined the volume of misinformation covered between February 1 and March 23, 2020, in two key types of sources. First, we looked at mainstream news, such as the *New York Times* or *USA Today* (MediaCloud “Top” Sources). Second, we examined explicitly conservative, right-leaning outlets, such as Fox News or Breitbart (Media Cloud “Right” Sources). For more details about the types of sources in each category, please see the online Appendix.

Survey data for this study come from Wave 63.5 of Pew's American Trends Panel (ATP). Panelists were invited to participate in ATP's nationally representative online panel from several probability-based surveys, from Winter 2014 to Fall 2019. Wave 63.5 was fielded between March 10 and March 16, 2020. Ipsos (on behalf of Pew) invited all 11,028 remaining panelists to participate, 8,914 of whom ultimately completed the survey.

All data used in this study are publicly available (Pew Research Center, 2020). Please consult the online Appendix for additional information about these data, including detailed information about how we measured key dependent and independent variables in the survey study.

## Results and Discussion

### Right-Leaning Media Were More Likely to Discuss COVID-Related Misinformation in Early March

Figure 1 plots news media stories in right-leaning and mainstream outlets, in the time period beginning on February 1 until March 23, several days after our survey data were collected. The patterns are clear: right-leaning outlets, such as Fox News, dedicated 3,839 stories that reference misinformation about COVID-19 in that time period, while mainstream outlets highlighted misinformation considerably less frequently (1,541 stories). Over-time patterns also highlight that misinformation spiked in early March, and the gulf in misinformation widened as the novel coronavirus continued to spread in March.

### More than One in Three Americans Endorse COVID-Related Misinformation

**Figure 2.**
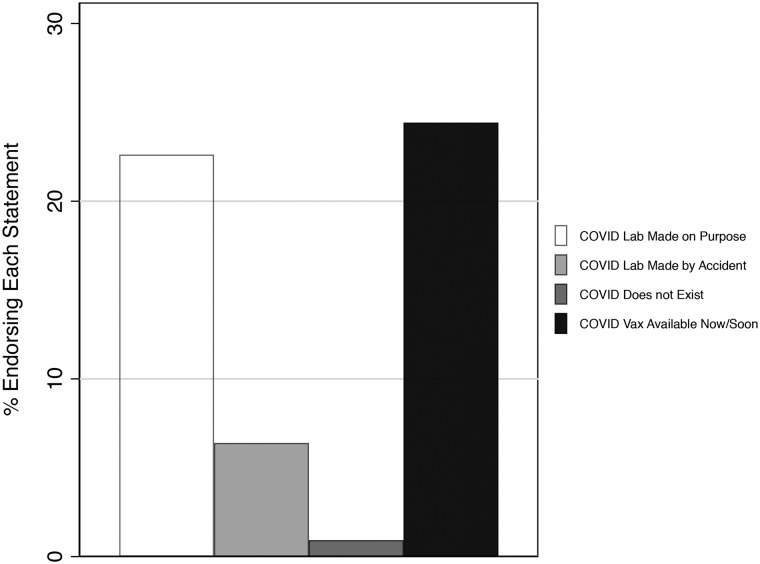
COVID-19 misinformation endorsement (March 10–16, 2020).

Note: Bars correspond to the weighted percentage of survey respondents in Pew's nationally representative ATP Wave 63.5 Survey (N = 8,914). Misinformation indicators are derived from responses to two questions. First, respondents were asked if they think “it is most likely that the current strain of the coronavirus” was either “developed intentionally in a lab,” “made accidentally in a lab,” “came about naturally,” or “does not exist.” From this, we created three dichotomous variables taking on values of 1 if respondents believe that COVID-19 was lab created (1) accidentally, (2) on purpose, (3) or if they believe that the virus does not exist. Respondents were also asked whether or not a COVID-19 vaccine is available “now,” “in the next few months,” “in a year or more,” or that “it is not possible to create a vaccine.” From this, we created a fourth dichotomous indicator, with those indicating that the vaccine is available now or will be available in the next few months scored as being misinformed.

Figure 2 displays the percentage of survey respondents in Pew's nationally representative American Trends Panel who endorsed COVID-related misinformation, between March 10 and March 16. The results suggest that more than one in five Americans (22%) believe that COVID-19 was purposefully created in a lab, and nearly one in four (24%) believe that a coronavirus vaccine exists now or will exist within the next few months. Fewer believe that COVID-19 was made in a lab by accident (7%) or that the virus does not exist at all (<1%). Overall, we find that more than one-third of Americans (38%) endorse at least one of these misinformed statements.

### Right-Leaning Media Viewers Are More than Twice as Likely to Endorse COVID-Related Misinformation

**Figure 3.**
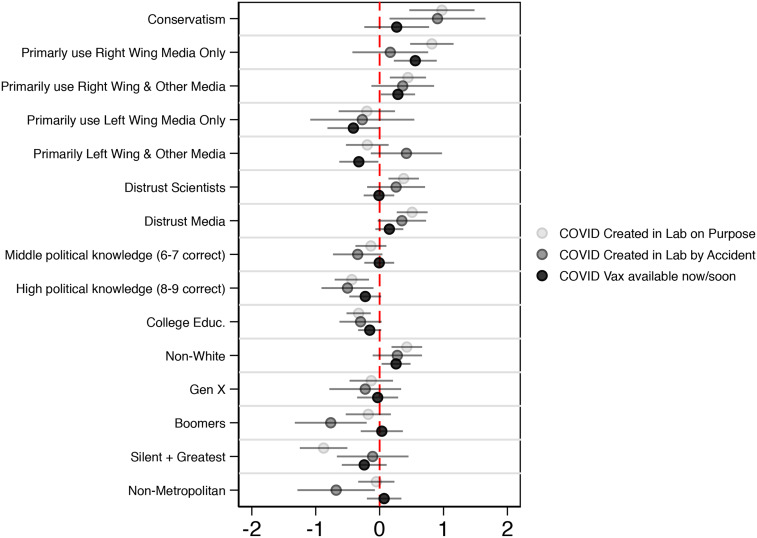
Correlates of misinformation endorsement (March 10–16, 2020).

Note: Logistic regression parameter estimates presented (shaded circles), with 95 per cent confidence intervals extending out from each one (N = 6,266). Note that, in order to avoid endogeneity concerns, antiscientist and antijournalist attitudes were measures prior to Wave 63.5 (ATP Wave 40, Fall 2019). Due to panel attrition, we lose N = 1,914. Note also that we do not include the “COVID-19 does not exist” misinformation indicator in these analyses, as fewer than 1 per cent of the sample endorsed this view (N = 56). Additional information about the ATP sample, how each control variable in the model was measured, and how we address potential endogeneity concerns can be found in the Methods section in the online Appendix. All data are weighted.

Figure 3 plots the association between survey respondents’ partisan news consumption habits and misinformation endorsement; this estimate controls for political, social, and demographic factors that might also influence misinformation endorsement. Circles falling to the right of the dashed red line indicate a positive relationship between a variable and endorsing a particular piece of misinformation. Circles falling to the left indicate a negative relationship. When the lines extending out from each circle do not intersect with the dashed red line, that relationship is statistically significant. For more technical information about the results of the logistic regression models used to produce this figure, please consult the Methods in the online Appendix.

We find that both people who solely (row 2 in Figure 3) or sometimes (row 3) consume right-leaning media were significantly more likely to believe that COVID was purposefully made in a lab, and that a COVID vaccine exists now (or will exist soon). However, we find no evidence that right-leaning news consumption was significantly associated with believing that COVID was accidentally lab-made. According to our models, while just 17 per ceny of people who primarily consume left-leaning media believed that COVID was purposefully lab-made, nearly double that number (34%) of exclusive right-leaning news consumers believed the same. Similarly, while 17 per cent of left-leaning news consumers believed that a COVID-19 vaccine already (or will soon) exist, more than double (35%) believed this claim among right-wing news consumers.

### Misinformed Americans Think that the CDC Exaggerated COVID's Health Risks

**Figure 4.**
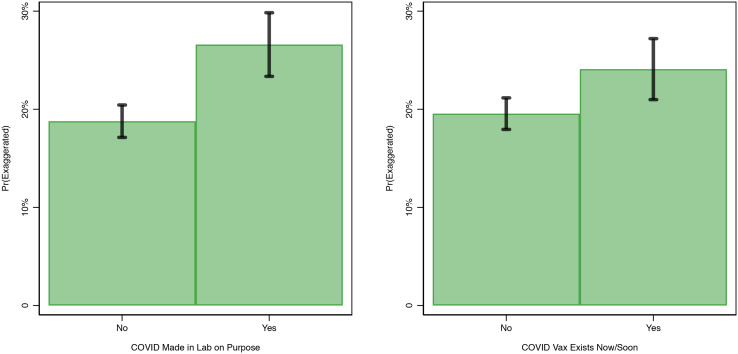
The effect of misinformation endorsement on anti-CDC attitudes (March 10–16, 2020).

Note: N = 8,568. Predicted probabilities (bars), with 95 per cent confidence intervals (lines). The outcome variable in this analysis is an indicator of whether or not respondents think that the CDC “greatly” or “slightly” exaggerated COVID-19's health risks. Probabilities were calculated based on the results of a logistic regression model, holding all covariates at their sample means. The model controls for all factors displayed in Figure 2, with the exception that we remove antiscientist and antijournalist views from these models (due to concerns of a lack of conceptual distinctness between these variables and the outcome variable). As a result, the valid N for this model is larger than the model results presented in Figure 2. Note also that we again exclude the “COVID-19 does not exist” variable from these analyses (see the note accompanying Figure 2). Additional information about how we measured the outcome variable in this analysis, as well as all independent variables, can be found in the “Online Methods” section of the online Appendix. All data are weighted.

Finally, Figure 4 plots (as green bars) the likelihood that people who endorse either one of the two pieces of COVID-related misinformation that we found to be influenced by right-leaning media viewership also report that the CDC is exaggerating COVID's public health harms risks. Here, too, 95 per cent confidence intervals are marked with black lines.^1^ Alarmingly, we find that people who believe that COVID was purposefully lab-created (26%) are significantly more likely to believe that the CDC is exaggerating the health risks of the virus than those who do not (19%). Similarly, people who believe that a vaccine already exists (24%), compared to those who do not (20%), are significantly more likely to distrust claims from the CDC.

## Conclusion

Our analyses suggest a clear relationship between right-leaning media consumption and pandemic-related public health beliefs. Right-leaning outlets were more likely to make inaccurate claims about the origins and treatment of COVID-19, and people who self-reported consuming more right-leaning news were subsequently more likely to express misinformed views. In turn, misinformed individuals were more likely to think that public health experts over-estimated the severity of the pandemic.

These findings must be interpreted with caution. While right-leaning media were more likely to discuss COVID-19 misinformation, we cannot observe directly whether people consumed specific stories that contained COVID-19 misinformation. Neither can we definitively disentangle the effects of factors, political ideology for example, that might encourage both right-leaning news consumption and misinformation endorsement. What we can say with confidence, however, is that we find a strong correlation between right-leaning media consumption and misinformation endorsement—one that holds even when we adjust for respondents’ ideological leaning and other social and demographic factors. We welcome experimental and longitudinal efforts that will replicate and expand upon the results presented here.

## Note

1 Again, for information on how we calculate these quantities, please review the online Appendix.

## Supplementary material

For supplementary material accompanying this paper visit https://doi.org/10.1017/S0008423920000396. click here to view supplementary material
