# Supplementary material for: How Right-Leaning Media Coverage of COVID-19 Facilitated the Spread of Misinformation in the Early Stages of the Pandemic in the U.S
Source: Can J Polit Sci. 2020 May 1:1–8. doi: 10.1017/S0008423920000396 (PMC7251254; doi:10.1017/S0008423920000396)
Supplement: Supplementary file 1 [file S0008423920000396sup001.docx]

Supplemental Materials for: How Right-Leaning Media Coverage of COVID-19 Facilitated the Spread of Misinformation in the Early Stages of the Pandemic

**Table S1. Full Model Output for Results used to Build Figure 3**

|  | COVID Made in Lab on Purpose | COVID Made in Lab by Accident | COVID Vaccine Exists Now/Soon |
| --- | --- | --- | --- |
|  |  |  |  |
| Conservatism | 0.98* | 0.91* | 0.27 |
|  | (0.26) | (0.38) | (0.26) |
| Primarily use Right Wing Media Only | 0.82* | 0.17 | 0.56* |
|  | (0.17) | (0.30) | (0.17) |
| Primarily use Right Wing & Other Media | 0.44* | 0.36 | 0.29* |
|  | (0.14) | (0.25) | (0.14) |
| Primarily use Left Wing Media Only | -0.20 | -0.27 | -0.41* |
|  | (0.22) | (0.42) | (0.21) |
| Primarily Left Wing & Other Media | -0.19 | 0.42 | -0.33* |
|  | (0.17) | (0.28) | (0.16) |
| Distrust Scientists | 0.38* | 0.26 | -0.01 |
|  | (0.12) | (0.23) | (0.12) |
| Distrust Media | 0.51* | 0.35+ | 0.15 |
|  | (0.12) | (0.19) | (0.11) |
| Med. Political Knowledge | -0.14 | -0.34+ | -0.01 |
|  | (0.12) | (0.20) | (0.12) |
| High Political Knowledge | -0.43* | -0.50* | -0.22+ |
|  | (0.14) | (0.21) | (0.13) |
| College Educated | -0.33* | -0.30+ | -0.15+ |
|  | (0.10) | (0.17) | (0.09) |
| Non-White | 0.43* | 0.28 | 0.26* |
|  | (0.12) | (0.20) | (0.12) |
| Gen X | -0.13 | -0.22 | -0.03 |
|  | (0.17) | (0.29) | (0.16) |
| Boomers | -0.18 | -0.76* | 0.03 |
|  | (0.18) | (0.29) | (0.17) |
| Silent + Greatest | -0.88* | -0.11 | -0.24 |
|  | (0.19) | (0.29) | (0.18) |
| Non-Metropolitan | -0.05 | -0.68* | 0.07 |
|  | (0.14) | (0.31) | (0.14) |
| Constant | -1.93* | -3.14* | -1.32* |
|  | (0.24) | (0.40) | (0.22) |
| N | 6,829 | 6,829 | 6,829 |

* p < 0.05, + p < 0.10; *two-tailed*

*Note.* Logistic regression parameter estimates presented with standard errors in parentheses. Please refer to the caption accompanying Figure 3 in the main text for additional information about these models.

**Table S2. Full Model Output for Results Used to Build Figure 4 in the Main Text**

| DV = Think CDC is Exaggerating COVID Risks |  |
| --- | --- |
|  |  |
|  |  |
| Misinfo: COVID Lab Made (Purpose) | 0.47* |
|  | (0.11) |
| Misinfo: COVID Lab Made (Accident) | 0.24 |
|  | (0.19) |
| Misinfo: COVID Vax. Available Soon/Now | 0.28* |
|  | (0.11) |
| Conservatism | 0.88* |
|  | (0.26) |
| Primarly use Right Wing Media Only | -0.48* |
|  | (0.17) |
| Primarily use Right Wing & Other Media | -0.16 |
|  | (0.13) |
| Primarily use Left Wing Media Only | -0.44* |
|  | (0.22) |
| Primarily Left Wing & Other Media | -0.56* |
|  | (0.14) |
| College Educated | -0.45* |
|  | (0.09) |
| Non-White | 0.07 |
|  | (0.11) |
| Gen X | 0.02 |
|  | (0.15) |
| Boomers | -0.06 |
|  | (0.15) |
| Silent + Greatest | -0.51* |
|  | (0.17) |
| Constant | -1.54* |
|  | (0.21) |
| N | 8,568 |

* p < 0.05, + p < 0.10; *two-tailed*

*Note.* Logistic regression parameter estimates presented with standard errors in parentheses. Please refer to the caption accompanying Figure 3 in the main text for additional information about these models.

**ONLINE METHODS**

**Content Analysis Data & Methods**

To examine the content of the news media environment surrounding COVID-19 misinformation, we have turned to the [MediaCloud](about:blank#/Home) Explorer, a tool designed to track news media outlets designed by the [MIT Center for Civic Media](about:blank) and the [Berkman Klein Center for Internet and Society at Harvard University](about:blank).

For our purposes in this paper, our goal is not to provide a detailed content analysis of the news media coverage of the novel coronavirus. Instead, our goal is to compare the volume of coverage of common misinformation about COVID-19 in two types of sources: right wing media and traditional, non-ideological news outlets. As a result, we compare two news outlet collections on MediaCloud. First, we look at the [U.S. Top Sources 2018](about:blank#/collections/186572516) (collection #186572516), which includes the most prominent outlets in the U.S., based on a Pew Research Center report published in 2019. It includes sources such as the New York Times, Washington Post, Wall Street Journal, USA Today, or Politico. Based on the Pew Research Center reports, these are the sources that most Americans trust and consume. Second, we examine the [Right Wing Sources](about:blank#/collections/9360524) (collection #9360524), which includes Fox News, Fox Business, Fox Nation, Breitbart, Rush Limbaugh, and other conservative outlets that tend to be trusted by those on the political right in the U.S. MediaCloud classified the sources as right wing based on relative number of tweets of election story urls by Clinton vs. Trump retweeters during the 2016 presidential election cycle.

Our search protocol was the following: ("coronavirus" or "covid") and ("made in a lab" OR "Big Pharma" OR "George Soros" OR "hoax" OR "conspiracy" OR "bioweapon" OR "not real" OR "existing vaccine")and was designed to capture common misinformation about COVID-19, such as that it is a hoax and does not exist, that it is a Chinese bioweapon, that the vaccine already exists but pharmaceutical companies won’t release it, and that it is a plot by the financier George Soros to destroy capitalism in the West. Our approach, of course, might miss other forms of misinformation about COVID, but our goal was to capture the most common misinformation. Furthermore, out approach does not analyze content in detail, and might miss “debunking” stories. However, given that research demonstrates the harm of repeating misinformation, even in the case of debunking, we are not worried about that caveat here.

We search the media for the terms outlined above for the time period of February 1, 2020 to March 23, 2020. That encompasses several weeks prior to the Pew survey data used in our analysis and a week after the survey.

**Survey Data**

Survey data for this study come from Wave 63.5 of Pew’s American Trends Panel (ATP). Panelists were invited to participate in ATP’s large and nationally representative online panel from several probability-based surveys; including three dual-frame phone surveys (recruited via cell phone and landline random digit dial; combined N = 19,922), and two address-based samples (combined N = 15,596), administered from Winter 2014 to Fall 2019.

Wave 63.5 was fielded between March 10 - March 16, 2020. Ipsos (on behalf of Pew) invited all 11,028 remaining panelists to participate, 8,914 of whom ultimately completed the survey (RR1 = 81%; cumulative response rate = 4.5%). All interviews were conducted online. Respondents lacking proper hardware or software were provided with an internet-accessible tablet.

To account for potential deviations between the final sample and the target population, data were weighted on the basis of age, gender, educational attainment, race, region, party identification, and several other factors (and combinations of factors). All data used in this study is publicly available. Additional technical information about ATP can be found at Pew’s website (see: Melia & Picco 2020).

**Measures**

*Misinformation Endorsement.* The primary outcome variables in our survey analyses – and the key independent variable in the second stage of our survey analyses – are four binary indicators of whether or not respondents endorse COVID-19 misinformation. These indicators are derived from responses to two questions. First, respondents were asked if they think “it is most likely that the current strain of the coronavirus” was either “developed intentionally in a lab,” “made accidentally in a lab,” “came about naturally,” or “does not exist.” From this, we created three dichotomous variables taking on values of 1 if respondents believe that COVID-19 was lab created (accidentally, on purpose) or if they believe that the virus does not exist.

Respondents were also asked whether or not a COVID-19 vaccine is available “now,” “in the next few months,” “in a year or more,” or that “it is not possible to create a vaccine.” From this, we created a fourth dichotomous indicator; with those indicating that the vaccine is available now, or will be available in the next few months scored as being misinformed.

Figure X in the main text summarizes the (weighted) distribution of misinformation endorsement on these four indicators. As the figure suggests, misinformation about COVID-19 is pervasive. More than a fifth of Americans believe that the disease was intentionally created in a lab, and nearly a quarter believe that a vaccine will exist in the near future. Note that, because less than 1% (N = 56) respondents endorsed the view that COVID-19 does not exist, we opted to exclude that indicator in our analyses.

*Distrust in Public Health Officials.* The key outcome variable in the second half of our survey analyses is an indicator of whether or not Americans trust information from public health officials about the severity of COVID-19. Respondents were asked whether or not “public health officials at the CDC” either “greatly exaggerated [COVID-19’s] risks,” “slightly exaggerated the risks,” “gotten the risks about right,” “not taken the risks quite seriously enough,” or “not taken the risks seriously at all.” We fashioned the resulting scores into a binary indicator of whether or not respondents perceived either great or slight exaggeration.

*Partisan News Consumption.* Consistent with Pew’s conventional practice, we measure respondents partisan news consumption habits by combining information -- assessed in Wave 57 of the ATP -- about respondents’ partisanship (i.e., whether they are self-identified Democrats or Republicans) and self-reported media exposure to left-leaning (e.g., MSNBC, New York Times, NPR), right-leaning (e.g., Rush Limbaugh, Fox News, Breitbart), and “mixed” audience outlets (e.g., Wall St. Journal). See Pew (2020) for additional information about how the organization determined each outlet’s partisan lean. We combine this information into four dichotomous indicators of whether or not respondents are Republicans (Democrats) who consume either mostly right-leaning (left-leaning) news, or a mix of different sources.

*Controls.* Our models account for a variety of different factors that could alternatively explain why people might endorse misinformation about COVID-19, and/or explain distrust in the CDC’s expressed levels of concern about the virus.

First, we control for respondents’ *political ideology*; a standard five-point measure (ranging from “Very Liberal” to “Very Conservative”), denoting respondents’ self-placement on a left-right ideological continuum. For consistency, we rescaled this variable (and all other variables) to range from 0-1. We include this as a control in our model, as previous research suggests that self-identified ideological conservatives may be more likely to endorse misinformation about public health, and distrust public health experts (e.g., Jost 2003; Gauchat 2012; Blank & Shaw 2015; Nisbet et al., 2015 Joslyn & Sylvester 2017; Motta 2018; Jost et al., 2018).

Next, we control for respondents’ *distrust in journalists and scientific authorities;* both of which have been shown to increase Americans’ receptivity to conspiratorial and factually inaccurate claims about public health (e.g., Kata 2010; Motta, Callaghan, & Sylvester 2018; MacFarlane et al., 2020; Stecula, Kuru, & Jamieson 2020).

In ATP Wave 40, respondents were asked to rate the extent to which they are confident that various individuals and institutions tend to “act in the best interests of the public.” Respondents could respond that they hold “a great deal,” “a fair amount,” “not too much,” or “no confidence at all” in “scientists” and either “the news media” (on two out of four randomly-assigned survey ballots), or “journalists” (on the remaining two ballots).

We scored respondents as expressing distrust in either group if they indicated “not too much” or “no confidence at all” in either group; noting that we combined the latter two targets (i.e., those focused on the media) into a single measure of distrust in journalists.

Note that, although ATP Wave 63.5 did ask respondents questions about their levels of trust in scientific and media authorities, we might be concerned about potential endogeneity issues. That is, because the questions were administered at the same time, we cannot be certain whether distrust in media/scientists facilitates misinformation endorsement, or *vice versa*.

Because the items we use were administered in ATP Wave 40 (Fall 2018), we circumvent this potential issue (i.e., because attitudes were assessed more than a year before the public was introduced to scientific and journalistic rhetoric concerning COVID-19). However, one downside of this approach is that we lose N = 1,914 cases as a result of panel attrition. Consequently, models studying the effects of partisan news media on misinformation endorsement contain N = 6,266 valid responses.

Of course, this approach cannot circumvent a different endogeneity concern; i.e., the possibility that our outcome variable in the models assessing the relationship of misinformation on trust in scientific authorities is conceptually not distinct from the distrust in scientific authorities variable. To alleviate this concern, and to perform these analyses on as many W63.5 respondents as possible, we removed both W40 indicators of distrust in scientific and journalistic authorities from the model.

Next, we control for respondents’ levels of *political knowledge* based on their performance on a short, nine-item civics knowledge quiz embedded in Wave 57 of the ATP. Note that, because all participants in Wave 63.5 also participated in Wave 57, we do not report any missing data on this variable; even though it is a lagged indicator of political knowledge. Consistent with Pew’s conventional practice, we trichotimized knowledge scores to reflect low (0-5 questions correct), medium (6-7 questions correct), and high (8-9 questions correct) knowledge. We control for political knowledge given the possibility that people who are better *informed* about politics and current events may be more likely to be *mis-informed* about related issues (e.g., Kuklinski et al., 2000; Pennycook & Rand 2019; although see: Miller Saunders & Farhart 2016; Kahan 2017).

Finally, our models control for respondents’ educational attainment (a binary indicator of whether or not respondents completed college), race (a binary indicator of whether or not respondents are Non-Hispanic and White), and metropolitan status (a binary indicator of whether or not respondents *do not* live in a major metropolitan area). We found it particularly useful, conceptually, to control for “non-metro” status, as individuals living in more sparsely populated areas may be less concerned about community transmission of COVID-19.

**Methods References**

Blank, J. M., & Shaw, D. (2015). Does partisanship shape attitudes toward science and public policy? The case for ideology and religion. *The ANNALS of the American Academy of Political and Social Science*, *658*(1), 18-35.

Kahan, D. M. (2017). Misconceptions, misinformation, and the logic of identity-protective cognition.

Kata, A. (2010). A postmodern Pandora's box: anti-vaccination misinformation on the Internet. *Vaccine*, *28*(7), 1709-1716.

Kuklinski, J. H., Quirk, P. J., Jerit, J., Schwieder, D., & Rich, R. F. (2000). Misinformation and the currency of democratic citizenship. *Journal of Politics*, *62*(3), 790-816.

Joslyn, M. R., & Sylvester, S. M. (2019). The determinants and consequences of accurate beliefs about childhood vaccinations. *American Politics Research*, *47*(3), 628-649.

Jost, J. T., Glaser, J., Kruglanski, A. W., & Sulloway, F. J. (2003). Political conservatism as motivated social cognition. *Psychological bulletin*, *129*(3), 339.

Jost, J. T., van der Linden, S., Panagopoulos, C., & Hardin, C. D. (2018). Ideological asymmetries in conformity, desire for shared reality, and the spread of misinformation. *Current opinion in psychology*, *23*, 77-83.

Merkley, Eric. "Anti-Intellectualism, Populism, and Motivated Resistance to Expert Consensus." (2020). *Forthcoming at Public Opinion Quarterly*

Miller, J. M., Saunders, K. L., & Farhart, C. E. (2016). Conspiracy endorsement as motivated reasoning: The moderating roles of political knowledge and trust. *American Journal of Political Science*, *60*(4), 824-844.

Motta, M., Callaghan, T., & Sylvester, S. (2018). Knowing less but presuming more: Dunning-Kruger effects and the endorsement of anti-vaccine policy attitudes. *Social Science & Medicine*, *211*, 274-281.

Nisbet, E. C., Cooper, K. E., & Garrett, R. K. (2015). The partisan brain: How dissonant science messages lead conservatives and liberals to (dis) trust science. *The ANNALS of the American Academy of Political and Social Science*, *658*(1), 36-66.

Pennycook, G., & Rand, D. G. (2019). Lazy, not biased: Susceptibility to partisan fake news is better explained by lack of reasoning than by motivated reasoning. *Cognition*, *188*, 39-50.
